# Evaluation of Bowel Monitoring and Escalation Practices for Clozapine-Related Constipation in a Women’s Medium Secure Forensic Setting

**DOI:** 10.1192/bjo.2026.11163

**Published:** 2026-06-30

**Authors:** Huma Khan, Matthew Manton, Isaura Gairin

**Affiliations:** 1 Midlands Partnership University NHS Foundation Trust, Stafford, United Kingdom; 2 Tees, Esk and Wear Valleys NHS Foundation Trust, York, United Kingdom; 3 South West Yorkshire Partnership Teaching NHS Foundation Trust, Wakefield, United Kingdom

## Abstract

**Aims:**

Clozapine-induced gastrointestinal hypomotility is a common and potentially fatal adverse effect, yet bowel monitoring and timely escalation remain inconsistently implemented despite national guidance. Following the removal of an electronic prescribing bowel-monitoring prompt in a medium secure women’s forensic service, concerns arose regarding reduced detection of constipation and increased patient safety risk.

This project aimed to evaluate baseline bowel monitoring and escalation practices for patients prescribed clozapine, explore staff and patient perspectives on existing monitoring systems, and assess the impact of introducing a structured paper-based bowel monitoring intervention on documentation quality and clinical escalation.

**Methods:**

A mixed-methods quality improvement project was conducted between April and August 2025 in the Women’s Pathway at Newton Lodge. Baseline practice was assessed via retrospective review of ward documentation and Clinical Team Meeting (CTM) notes for all patients prescribed clozapine. Staff questionnaires explored perceptions of monitoring systems, safety, and usability. Semi-structured patient interviews examined acceptability and communication preferences. An iterative paper-based bowel monitoring chart was introduced, refined following staff feedback, and re-audited against national standards.

**Results:**

Seventeen patients prescribed clozapine were included in the documentation audit. Baseline recording of bowel status in the CTM notes occurred on approximately 10% of inpatient days. Seventeen staff responses were obtained across nursing and medical disciplines. Fifteen staff members (88%) reported that the previous electronic prescribing prompt improved patient safety, and the majority perceived increased risk following its removal. Initial uptake of a detailed paper bowel monitoring chart was low, with completion rates of approximately 25% over four weeks. Following simplification of the chart, completion increased to nearly 100% and was sustained on re-evaluation. Documentation of bowel status in the CTM notes improved from approximately 10% pre-intervention to almost 100% post-intervention. Escalation and documented clinical review for patients with more than 48 hours without bowel movement increased from approximately 60% at baseline to 100% following intervention. Five patient interviews demonstrated general acceptability of bowel monitoring, with preference for simple, discreet questioning methods.

**Conclusion:**

A simplified bowel monitoring intervention significantly improved documentation and escalation of clozapine-related constipation. Integration into electronic prescribing systems, alongside staff training and standardised escalation thresholds, is recommended to ensure sustainable patient safety improvements.

